# Effect of Moxibustion on the Intestinal Flora of Rats with Knee Osteoarthritis Induced by Monosodium Iodoacetate

**DOI:** 10.1155/2020/3196427

**Published:** 2020-07-04

**Authors:** Yu Chen, Jiuheng Lv, Yejuan Jia, Ruiqing Wang, Zidi Zhang, Jingxuan Liu, Chunsheng Jia

**Affiliations:** Hebei University of Chinese Medicine, Shijiazhuang 050200, China

## Abstract

In this study, a knee osteoarthritis (KOA) rat model induced by monosodium iodoacetate (MIA) was used to study the effect of moxibustion on improving knee cartilage damage and its effect on the intestinal flora. The experimental rats were divided into the normal group (N), model group (M), moxibustion treatment group (MS), and diclofenac sodium treatment group (DS). After 4 weeks, cartilage pathological damage in the knee joint was evaluated using hematoxylin-eosin and safranin O-fast green staining analysis. ELISAs and Western blots were used to detect the expression levels of IL-1*β* and TNF-*α* in the serum and cartilage, respectively. The total DNA of the fecal samples was extracted and subjected to high-throughput sequencing of the V3-V4 region of the 16S rRNA gene to analyze the changes in the intestinal flora. In the model group, the cartilage was obviously damaged, the expression levels of IL-1*β* and TNF-*α* in the serum and cartilage were increased, and the abundance and diversity of the intestinal flora were decreased. Moxibustion treatment significantly improved the cartilage damage and reduced the concentration of inflammatory factors in the serum and cartilage. The high-throughput sequencing results showed that compared to the model group, the moxibustion treatment regulated some specific species in the intestinal microorganisms rather than the *α* diversity. In conclusion, our findings suggest that moxibustion treatment may work through two aspects in rats. On one hand, it directly acts on knee cartilage to promote repair, and on the other hand, it regulates the composition of the intestinal flora and reduces the production of inflammatory factors.

## 1. Introduction

Knee osteoarthritis (KOA) is one of the most common joint diseases, and one-third of people over 65 years old in China suffer from it according to epidemiological surveys [1]. KOA is generally considered to be a kind of degenerative cartilage damage, which has gradually been realized to be caused by chronic low-grade inflammation as the understanding of the disease has increased in the past decade [2]. Local tissue damage caused by obesity, advanced age, and genetics can produce a large quantity of damage-associated molecular patterns (DAMPs), which can increase the production of inflammatory factors (such as interleukin-1*β* and tumor necrosis factor-*α*) in the cartilage of the knee joint through various inflammation-promoting effects. The increased inflammatory factors, which cause a chronic continuous low-grade systemic inflammatory environment, directly induce cartilage catabolism, leading to the failure of cartilage tissue repair and inflammation cycle and ultimately to cartilage loss and degeneration [3].

At present, increasing evidence has suggested that the intestinal flora plays an important role in diseases, including obesity, type 2 diabetes mellitus, and cancer [4]. Recent research [5] has shown that the emergence and development of systemic chronic low-grade inflammation is closely associated with the intestinal flora, and the composition of which may directly affect lipopolysaccharide (LPS) content to affect the innate immune system. In KOA, LPS is the key proinflammatory substance among microbial products and is associated with the severity of inflammation, pain, narrowing of the joint space, and osteophyte formation [2]. A large population-based cohort study on the intestinal microbiome of KOA patients [6] showed that the microbiome diversity is different in individuals with increased knee Western Ontario and McMaster Universities Osteoarthritis Index (WOMAC) pain scores, and the abundance of certain members of the intestinal flora (streptococci) is associated with increased knee pain. Therefore, the intestinal flora is a very promising potential target for relieving the severity of disease in patients with KOA and even a possible therapeutic target for osteoarthritis-related knee pain.

In the treatment of KOA, nonsteroidal drugs are commonly used for their anti-inflammatory activity and improving the disease process of patients, but the accompanying adverse reactions, such as gastrointestinal toxicity, limit their use [7]. Therefore, nondrug complementary and alternative therapies have gradually attracted the attention of physicians. Among them, moxibustion, as an important part of complementary and alternative medicine in the world, has become an option for the treatment of knee osteoarthritis due to its noninvasive, safe, and effective characteristics [8]. Recent studies have confirmed that moxibustion can have a protective effect on cartilage by reducing proinflammatory substances such as IL-1*β* and TNF-*α* in the synovial fluid of KOA rats [9] and can also alleviate intestinal inflammation by regulating intestinal flora [10].

However, in the KOA treatment process, the effect of moxibustion on the composition of the intestinal flora and the effect of the intestinal flora change on the treatment are not known. Therefore, this study aimed to construct a KOA rat model induced by MIA, analyze the effect of moxibustion on the intestinal flora of KOA rats by high-throughput sequencing, and then elaborate the mechanism by which moxibustion affects KOA from the perspective of the intestinal flora.

## 2. Materials and Methods

### 2.1. Experimental Animals and Groups

Thirty-six male Wistar rats (SPF grade, 12 weeks old, 230–280 g) were provided by the Hebei Experimental Animal Center (license number: SCXK (Ji) 2018-004). All rats were kept under standard conditions: temperature 25 ± 1°C, humidity 50%–70%, 12 h light/dark cycle, and free access to standard rat feed and water. All animal care and experiments were performed in accordance with procedures approved by the Animal Care and Use Committee of the Hebei University of Traditional Chinese Medicine (Animal Study Approval No. DWLL2018025), with best efforts made to avoid injury and pain. After 1 week of adaptive feeding, the rats were randomly divided into 4 groups (*n* = 9): normal group (N), model group (M), moxibustion treatment group (MS), and diclofenac sodium treatment group (DS).

### 2.2. Model Preparation and Therapeutic Intervention

**Figure 1:**
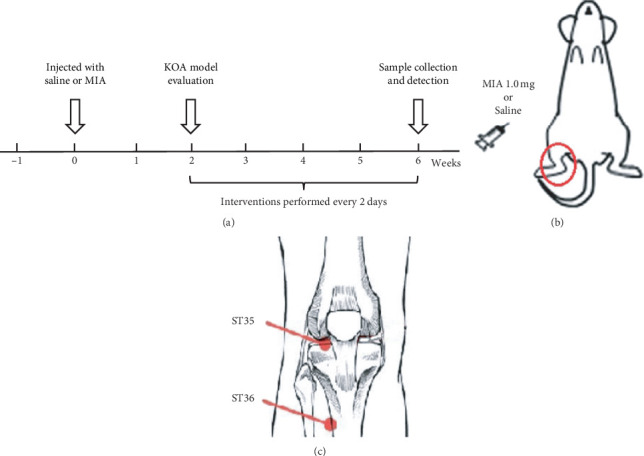
Model preparation and moxibustion treatment. (a) The preparation of the MIA-induced KOA rat model. (b) The location of MIA and saline injection. (c) The location of the acpoints used for moxibustion treatment.

According to the method described by Pitcher et al. [11], as shown in Figure 1, the rats in the N group were injected with saline (50 *μ*L) as a control, while the rats in other groups had KOA induced by injecting a physiological saline solution of MIA (1 mg in 50 *μ*L). MIA and saline were injected into the right posterior knee joint cavity by patellar ligament puncture. Two weeks later, one rat in each group was randomly selected for pathological examination of the knee joint cartilage. When there was no significant change in the cartilage of the knee joint in the control group and the group injected with MIA showed destruction of the cartilage surface, abnormal safranin O staining, and Mankin score (according to Table 1) of 4–7 points, the cartilage of the knee joint met the standard of moderate knee osteoarthritis, and the KOA rat model was successfully constructed [12]. After the model was successfully constructed, therapeutic intervention was performed. The rats in group M were subjected to KOA establishment without any subsequent treatment, simulating the natural recovery of KOA without any treatment. For the MS group, according to Ma et al. [13], the location of the treatment site is shown in Figure 1, where Dubi (ST35) is located in the depression outside the patellar ligament and Zusanli (ST36) is located at about 5 mm inferior of the capitulum fibulae and posterior-lateral to the hind-limb knee joint [14]. After KOA was established in the rats, the lit moxibustion strip (diameter 7 mm, length 12 cm) was placed 1-2 cm away from the treatment acupoint, the skin temperature “of” the acupoint was maintained at 43 ± 1°C, and the treatment lasted for 30 min [10]. In the DS group, rats were given diclofenac sodium sustained-release tablet solution (Futalin, Beijing Novartis Pharmaceutical Co., Ltd., CFDA approval number: H10980297) by gavage at a dose of 0.15 mg/100 g body weight, equivalent to the dose used for human treatment [15]. All treatments were performed every 2 days for 4 weeks, 14 times in total. After the treatment concluded, the feces of each group were collected by abdominal pressing. Then, using the method described by Miyamoto et al. [12], all rats were anesthetized by intraperitoneal injection of pentobarbital sodium (40 mg/kg), and knee joint and blood samples of each group were collected. Finally, the rats were killed by cervical dislocation.

### 2.3. Histopathological Examination of the Cartilage

The pathological damage of the knee joint cartilage was observed by hematoxylin-eosin staining and safranin O-fast green staining. Then, the samples were decalcified in 10% ethylenediaminetetraacetic acid (EDTA) solution for 14 days, dehydrated with ethanol, cleared with xylene, and paraffin embedded. The paraffin-embedded samples were sliced into sections with a thickness of 5 *μ*m. Hematoxylin-eosin (HE) staining and safranin O-fast green staining were used to observe the morphology and proteoglycan loss of the cartilage tissue, respectively. Subsequently, the staining of the cartilage samples was observed by optical microscopy (Olympus Co., Ltd., Tokyo, Japan), and the damage to cartilage tissue was evaluated by the modified Mankin scale (Table 1) [16].

### 2.4. Detection of the Cartilage Tissue Inflammatory Factors IL-1*β* and TNF-*α*

Western blotting was used to detect the relative expression of IL-1*β* and TNF-*α* in the cartilage of the distal femur. Fresh articular cartilage was frozen in liquid nitrogen immediately after removal and then used for the extraction of total protein. The concentration of the extracted protein was quantified by the BCA method. Subsequently, the protein samples were mixed with 5x loading buffer, denatured at 100°C for 5 min, separated by SDS-PAGE, and finally transferred to PVDF membranes (Millipore Corporation, Bedford, MA, USA). The protein samples were blocked with 5% skimmed milk for 1 h, incubated overnight at 4°C with the primary antibody, and then incubated on a shaking table at room temperature with the secondary antibody for 1 h. The optical density of the bands was observed with an Odyssey infrared fluorescence imaging system (LI-COR, USA) and quantitatively analyzed in ImageJ software. *β*-Actin was used as the internal control in this study. The primary antibodies used were as follows: IL-1*β* (ABclonal, A11369, 1 : 1000), TNF-*α* (Wuhan Sanying, 17590-1-AP, 1 : 1000), and *β*-actin (Servicebio, GB12001, 1 : 1000).

### 2.5. Detection of the IL-1*β*, TNF-*α*, and LPS Content in Serum

The enzyme-linked immunosorbent assay (ELISA) was used to detect the inflammatory factors IL-1*β* and TNF-*α* in the serum. After the rats were sacrificed, 3 ml of blood was collected through the abdominal aorta. Blood samples were then centrifuged for 15 min at 4°C and 3000 RPM/min in a high-speed chilled centrifuge (Thermo Fisher Scientific Co., Ltd., China) to collect the serum. According to the manufacturer's instructions, the concentration (pg/ml) of IL-1*β* and TNF-*α* in the serum was measured with the corresponding ELISA kit (IL-1*β*: Gemini, ek301b/3-96, China; TNF-*α*: Thermo Fisher Scientific, 88-7340-88, China). The LPS content in serum was evaluated using an endpoint chromogenic tachypleus amebocyte lysate kit for detection of bacterial endotoxin (ECK-082, Zhanjiang A&C Biological Ltd., China).

### 2.6. Fecal DNA Extraction and Sequencing of the 16S rRNA Gene V3-V4 Region

After 4 weeks of treatment intervention, 2 pellets of fresh feces were collected from each rat and stored in sterile tubes at −80°C until detection. In each group, the feces of 3 rats were randomly selected for high-throughput sequencing of the intestinal flora. All sequencing work was carried out by Biomarker Technologies, Beijing, China. A QIAamp DNA Mini kit (Qiagen, Hilden, Germany) was used to extract total bacterial genomic DNA from the stool samples. The universal primers 319F and 806R (319F: 5′ACTCCTACGGGAGGCAGCAG3′ and 806R: 5′GGACTACHVGGGTWTCTAAT3′) were used to amplify the V3-V4 region of the 16S rRNA gene. The PCR amplification was carried out in a GeneAmp PCR System 9700 thermal cycler (Applied Biosystems, Foster City, CA, USA). The conditions were as follows: an initial denaturation step at 95°C for 2 min, followed by 25 cycles of denaturation at 95°C for 30 s, annealing at 55°C for 30 s, and extension at 72°C for 30 s, and a final extension step at 72°C for 5 min. The amplified products were purified, quantified, and homogenized to form sequencing libraries. After the quality of the constructed library was tested and qualified, high-throughput sequencing was carried out using the Illumina HiSeq 2500 sequencing platform. The UCLUST analysis toolkit in QIIME software was used to perform a cluster analysis on all tags at a similarity level of 97%. The obtained Operational Taxonomic Units (OTUs) were classified based on the Silva (bacterial) taxonomy database. A Venn diagram is used to show the number of common and unique OTUs among samples, and the coincidence of OTUs among samples is intuitively shown [17]. The taxonomy structure of the samples at phylum and genus levels was drawn by using R software (ver. 3.1.0). For the *β* diversity, the differences between gut microbiota in the 4 groups was calculated by using weighted UniFrac algorithm and visualized by using principal coordinate analysis (PCoA) at the OUT level [18]. The software packages mothur (version v.1.30) permitted the determination of the Shannon, Simpson, Chao1, and ACE indices and rare faction curves for the alpha-diversity analysis, while R was used for plotting those curves [19]. Linear discriminant analysis effect size (LEfSe) analysis in multilevel species was used to determine potential bacterial biomarkers with statistical significance between different groups [20].

### 2.7. Statistical Methods

SPSS 22.0 software (version 22.0, IBM, Chicago, IL, USA) was used for statistical analyses. Measurement data are expressed as the mean ± standard deviation. If the characteristics of the data met the normal distribution, the *t*-test was used. If the data did not meet the normal distribution criteria, the rank-sum test was used. Intragroup comparisons were performed using one-way analysis of variance, and intergroup comparisons were performed using the LSD test. *P* < 0.05 was considered statistically significant.

## 3. Results

### 3.1. Moxibustion Treatment Significantly Improves the Pathological Damage of the Cartilage Tissue in the Knee Joint

**Figure 2:**
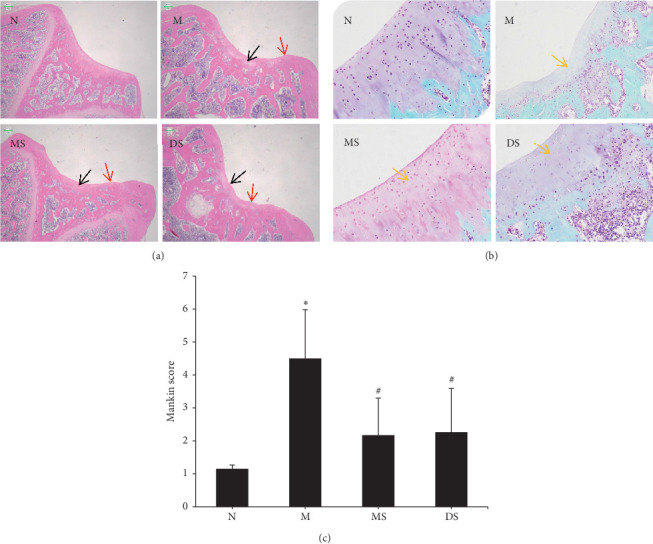
Histological analysis of cartilage samples (original magnification, ×40) from the distal end of the rat femur in rats treated with MS and DS for 4 weeks. N shows a normal sample structure. M shows strong inflammatory damage. MS shows a reduction in the extent and severity of the histological cell damage. DS shows a relatively normal structure. (a) Hematoxylin-eosin staining analysis (the red arrow represents the unevenness of the cartilage surface and the black arrow represents the loss of chondrocytes), (b) safranin O-fast green staining analysis (the yellow arrow represents the loss of the cell matrix), and (c) microscopic structure damage of the cartilage tissue according to the modified Mankin scale. ^*∗*^Compared with (N) *P* < 0.05; ^#^compared with (M)*P* < 0.05.

The main pathological characteristics of KOA are articular cartilage injury, osteophyte formation, and synovial hyperplasia, among which the degree of cartilage injury is the key determinant of KOA [21]. Therefore, the morphology of chondrocytes in the knee joints (Figure 2(a)) and the loss of glycoproteins in the chondrocyte matrix (Figure 2(b)) of rats in each group were evaluated using HE and safranin O-fast green staining.  Figure 2(c) shows the related Mankin scores for the knee cartilage tissue damage observed in each group of rats. The results indicate that there was no obvious abnormality of the cartilage tissue (Figures 2(a) and 2(b)) in the N group. In the M group, the integrity of the articular cartilage was severely damaged (Figure 2(a)), including unevenness of the cartilage surface, a disordered chondrocyte hierarchy, visible fibrosis, the loss and necrosis of a large number of chondrocytes, and a large number of cartilage pits. Furthermore, in group M, there was a wide range of decolorization in the cartilage matrix (Figure 2(b)), indicating that proteoglycan was markedly reduced. In comparison, in the MS and DS groups (Figure 2(a)), the roughness of the cartilage surface was reduced, and the quantity of chondrocytes was increased compared with that in the model group. Furthermore, deep matrix staining (Figure 2(b)) also showed that proteoglycan levels were less reduced in the MS and DS group samples. The Mankin score (Figure 2(c)) of the abovementioned articular chondrocyte destruction showed that the cartilage injury score of the M group was significantly higher than that of the N group (*P* < 0.05). The intervention treatment of the MS and DS groups significantly reduced the cartilage injury score (*P* < 0.05), and the difference between the scores of the two intervention treatments was not significant (*P* > 0.05).

**Figure 3:**
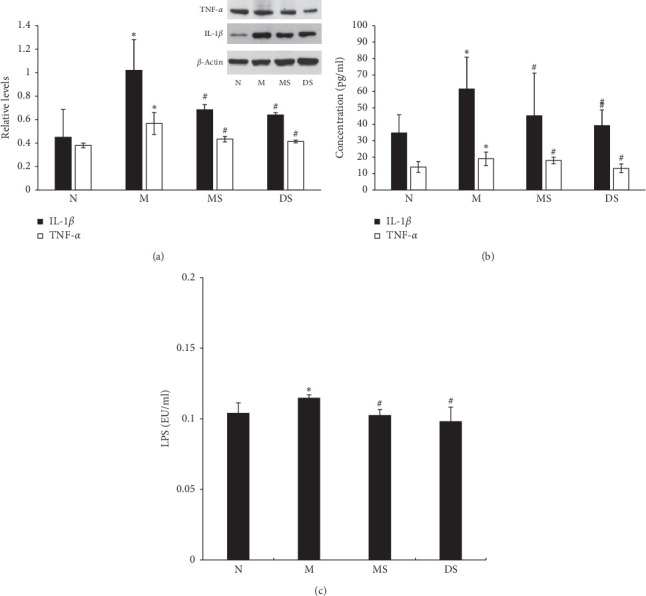
(a) The relative expression level of IL-1*β* and TNF-*α* in the cartilage. (b) The content of IL-1*β* and TNF-*α* in serum. (c) The content of LPS in serum. ^*∗*^Compared with (*N*) *P* < 0.05; ^#^compared with (M) *P* < 0.05.

The ELISA and Western blot results are shown in Figure 3. The Western blot results showed that the inflammation of the articular cartilage was significantly increased in group M.  Figure 3(a) shows that the relative expression levels of IL-1*β* and TNF-*α* relative to the internal control (*β*-actin) were 1.02 ± 0.26 and 0.57 ± 0.09, respectively, significantly higher than that of group N (0.45 ± 0.24 and 0.38 ± 0.02, *P* < 0.05). After moxibustion and diclofenac sodium treatment, the relative expression of these two inflammatory factors in the cartilage was significantly decreased (*P* < 0.05). In the MS and DS groups, IL-1*β* decreased to 0.68 ± 0.05 and 0.64 ± 0.02, and TNF-*α* decreased to 0.43 ± 0.02 and 0.41 ± 0.01, respectively. In addition, there was no significant difference in the IL-1*β* and TNF-*α* decrease between the two treatment groups (*P* > 0.05), indicating that the two treatment methods had a similar effect in alleviating the cartilage inflammation. For the above two inflammatory factors in the serum, the ELISAs showed a trend similar to that in the cartilage tissue. In group M, the IL-1*β* and TNF-*α* reached 61.45 ± 19.32 pg/ml and 18.98 ± 4.11 pg/ml, respectively, which was significantly higher than that in group N (34.68 ± 11.1 pg/ml and 13.99 ± 3.28 pg/ml, *P* < 0.05), indicating that there the severity of the systemic inflammation in group M was increased. Similarly, the two inflammatory factor levels in the MS group (IL-1*β*: 45.10 ± 25.95 pg/ml and TNF-*α*: 17.98 ± 1.96 pg/ml) and DS group (IL-1*β*: 39.19 ± 9.57 pg/ml and TNF- *α*: 13.19 ± 2.71 pg/ml) were significantly decreased (*P* < 0.05), and there was no significant difference between the two groups (*P* > 0.05). Due to the importance of the role in the systemic inflammation, the contents of LPS in serum were detected (Figure 3(c)). The results showed that the level of LPS in group M (0.115 ± 0.002 EU/ml) was significantly higher than that in group N (0.104 ± 0.007 EU/ml, *P* < 0.05). And after the treatment of moxibustion and diclofenac sodium, the LPS levels were significantly decreased to 0.103 ± 0.004 EU/ml in group MS (*P* < 0.05) and 0.098 ± 0.010 EU/ml in group DS (*P* < 0.05).

### 3.2. Moxibustion Treatment Changes the Composition and Diversity of Intestinal Flora in Rats

**Figure 4:**
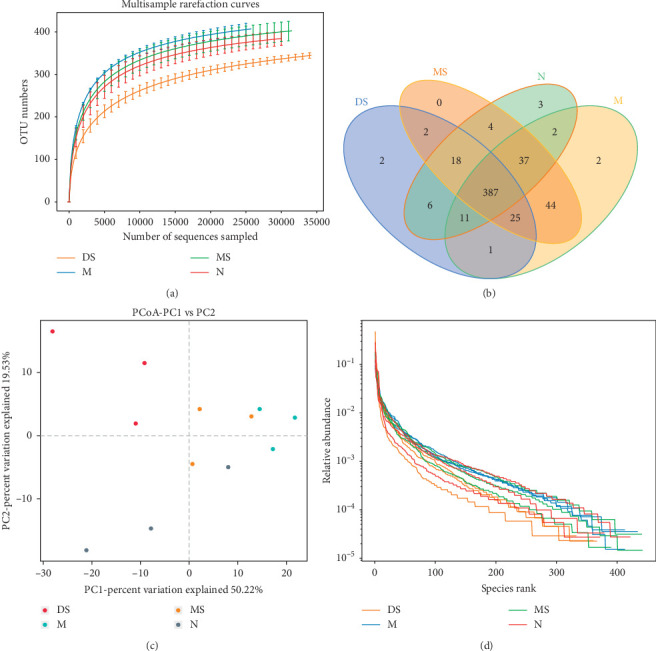
Analysis of the intestinal flora sequences in different groups. (a) Rarefaction curves. The horizontal coordinate shows the number of randomly selected sequence, and the vertical coordinate shows the number of OTUs based on the clustering of the sequencing lines. Each curve represents a sample, marked with different colors. (b) OTU Venn diagram. The numbers in overlapping parts between the different color patterns are the OTU amounts shared by the samples, and the numbers in nonoverlapping parts are the unique OTU amounts in each sample. (c) Weighted UniFrac PCoA analysis of the differences between the 4 groups. Points represent each sample separately; different colors represent different groups; the horizontal and vertical coordinates are the two characteristic values producing the greatest difference between samples, the horizontal coordinate represents PC1, the vertical ordinate represents PC2, and the percentage shows the main influence degree. (d) Rank-abundance curves. The abscissa is the number sorted by OTU abundance, and the ordinate is the relative abundance of the corresponding OTUs.

A total of 811779 high-quality sequences were obtained from 12 samples from 4 groups. Each sample produced at least 45629 reads, with an average of 67648. OTUs were obtained by clustering the obtained sequences at a 97% similarity level. The rarefaction curve (Figure 4(a)) shows that the new OTUs in each sample tended to increase gently as the sequencing number increased. This finding shows that the sequencing coverage of each sample was sufficient for data analysis. Then, the similarity and overlap of 544 OTUs identified at the genus level were analyzed by the Venn diagram (Figure 4(b)). Among them, 387 OTUs were thought to be the core components as appeared in all four groups. The number of unique OTUs in each group was 3 in group N, 2 in group M, 0 in group MS, and 2 in group DS. The total number of OTUs in the N group, M group, MS group, and DS group was 468, 509, 517, and 452, respectively. The number of OTUs in the MS group is the highest, and the number of OTUs in the M group is the lowest, suggests that OTUs in the MS group would be the most abundant.

The *β* diversity represents the diversity difference in the intestinal microbial community structure of rats between each group. It was analyzed by using weighted UniFrac principal coordinate analysis (PCoA; Figure 4(c)). Through a series of eigenvalue and eigenvector sorting, the most important elements and structures were extracted from multidimensional data, and the principal coordinate combination with the largest contribution rate was selected for analysis. PC1 is the first principal coordinate, representing 50.22% of the total flora, and PC2 is the vertical coordinate, representing 19.53% of the total flora. The results showed that the samples from group M and group N were significantly separated (*P*=0.005 for the PC1, and *P*=0.030 for the PC2), which indicates that the structure and composition of the intestinal flora in the KOA rat model had obvious changes. The DS group was far from the M group (*P*=0.001 for the PC1, and *P*=0.283 for the PC2) and followed the direction of the N group, which suggests that diclofenac sodium might improve the imbalance of the intestinal flora. However, the large distance between the DS group and the N group (*P*=0.174 for the PC1, and *P*=0.005 for the PC2) suggests that diclofenac sodium may have a negative regulatory effect on the intestinal flora, which is consistent with the results of previous studies [22]. The MS group was located between the M group and the N group and was relatively close to the N group (*P*=0.174 for the PC1, and *P*=0.039 for the PC2), which shows that moxibustion treatment restores part of the difference between the M and N groups and has a favorable regulatory effect on the intestinal flora.

Therefore, the *α* diversity of the intestinal flora in the four groups of rats was analyzed. The *α* diversity (including the index of Chao1, Shannon, Ace, and Simpson) was used to investigate bacterial species diversity of the intestinal flora. A statistical t-test was used to detect whether the index value between groups was significantly different [20]. Among the indices, the Chao1 and ACE indices reflect the richness of the flora and the Shannon and Simpson indices reflect the diversity of the flora. As shown in Table 2, the ACE index, Chao1 index, and Shannon index of M and MS groups tended to increase compared with the N group, while the DS group tended to decrease compared with the N group. For the Simpson index, in addition to the significant decrease in group M (*P* < 0.05), the index also showed a downward trend in group MS, while it slightly increased in group DS. The effect of moxibustion and diclofenac sodium on the *α* diversity of intestinal flora can be obtained from the changes in each group compared with that in group M. As shown in Table 2, compared with the M group, the treatment in the MS group did not significantly change the *α* diversity indices, while the treatment of the DS group changed all the *α* diversity indices significantly (*P* < 0.05). Furthermore, the rank-abundance curves (Figure 4(d)) showed that the abundance of OTUs in the moxibustion group had a more moderate slope and a wider distribution on the horizontal axis than other groups, which indicates that the moxibustion intervention induces a richer diversity and a more uniform distribution of bacterial composition than the other treatments tested.

### 3.3. Effect of Moxibustion Treatment on the Microbial Community Structure of the Intestinal Flora

**Figure 5:**
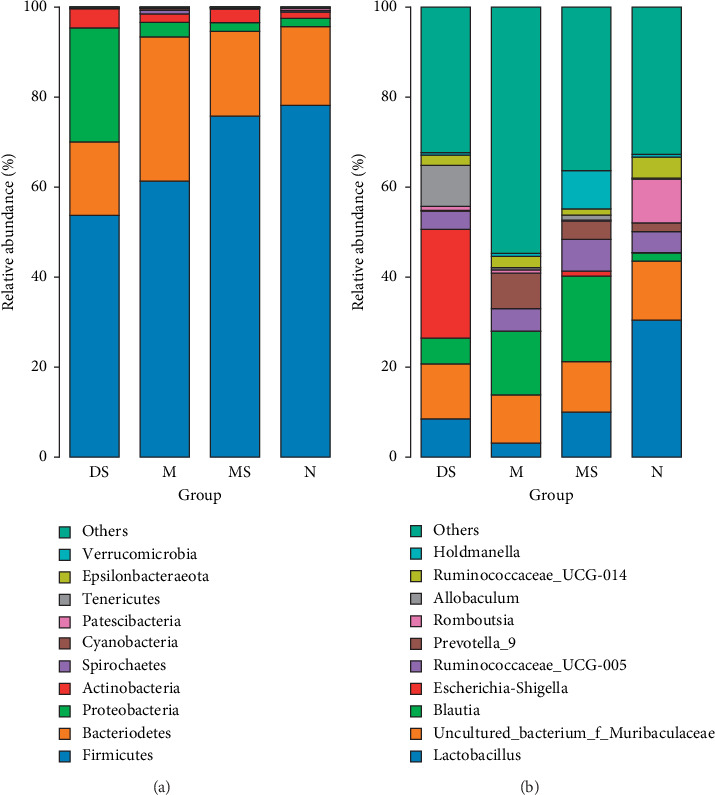
Analysis of the microbial composition at the phylum (a) and genus (b) levels.

The results of the analysis at the phylum level are shown in Figure 5(a). All the samples contained three main phyla: Firmicutes, Bacteroidetes, and Proteobacteria. Among them, in group M, the abundance of Firmicutes was decreased and that of Bacteroidetes was increased, which may also be a characteristic of the intestinal flora in patients with KOA. According to the data of the MS group, the 4-week moxibustion treatment increased the abundance of Firmicutes and decreased the abundance of Bacteroidetes, which made the composition similar to that of the N group. In contrast, in the DS group, the abundance of Firmicutes and Bacteroidetes was decreased, whereas that of Proteobacteria was markedly increased. Firmicutes are mainly composed of Gram-positive bacteria, while Bacteroidetes and Proteobacteria are mainly composed of Gram-negative bacteria. In addition, for the Firmicutes/Bacteroidetes, the ratio in the M group decreased significantly, while recovered in the MS group. For further discussion, we analyzed the species composition of the intestinal flora at the genus level (Figure 5(b)). The results showed that the abundance of *Lactobacillus* decreased and *Blautia* increased in group M compared with group N (normal rats). After moxibustion and diclofenac sodium treatment, the intestinal flora of the KOA model changed differently. Compared with that in the M group, the abundance of *Lactobacillus* and *Blautia* was increased in the MS group, whereas the abundance of *Lactobacillus* and *Escherichia-Shigella* was increased, and the abundance of *Blautia* was decreased in the DS group. In addition, *Escherichia-Shigella*, a typical pathogen of bacillary dysentery, was highly enriched in the DS group, reaching 24.16%, approximately 400 times higher than that in the normal group (0.06%).

**Figure 6:**
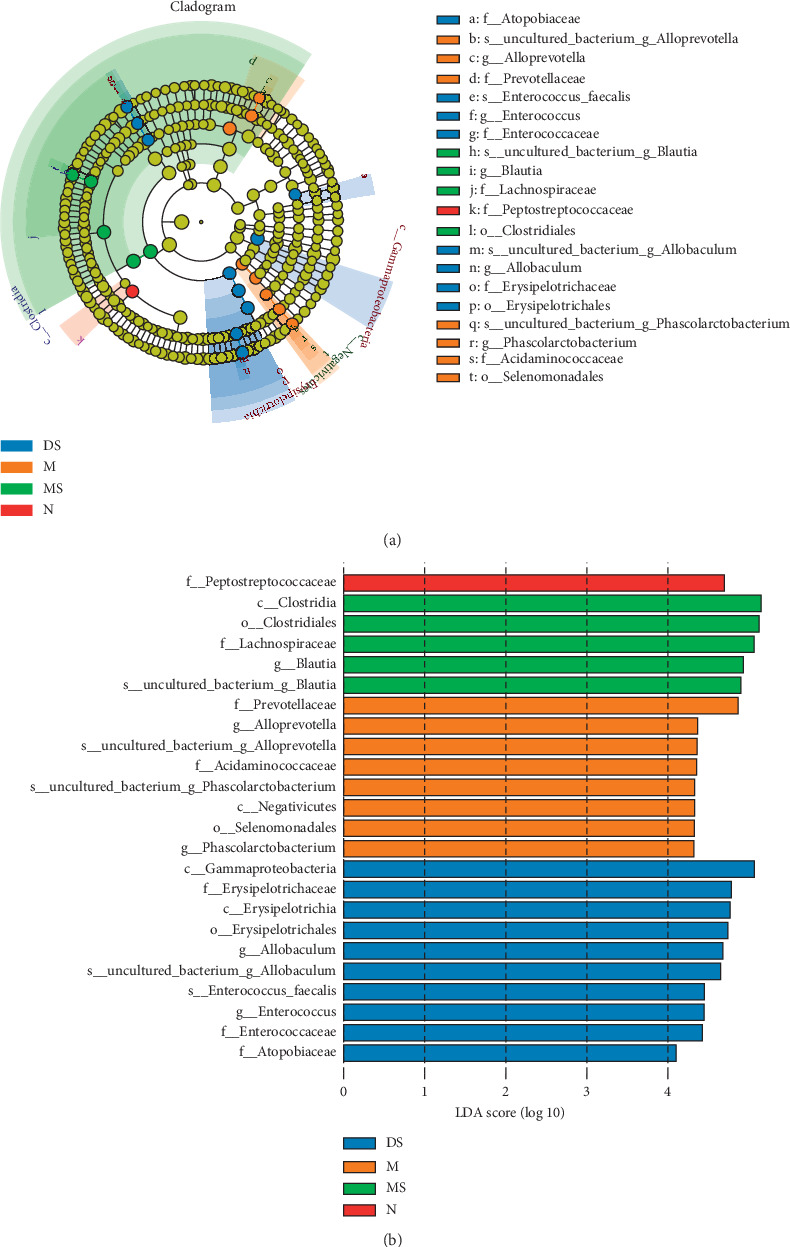
LEfSe (a) and LDA (b) analyses based on OTUs characterizing the microbiomes of the 4 groups. (a) The circle in the LEfSe cladogram radiating from inside to outside represents the classification level from the phylum to species; each small circle represents a classification at this level, and the diameter of the small circle is proportional to the relative abundance; the species without significant differences are uniformly colored yellow, and other species with different abundances are colored according to the group with the highest abundance of the species. Different colors represent different groups, and the different color nodes represent the microbial groups that play an important role in the groups represented by the color. (b) The graph shows the bacteria with an LDA score greater than 4.0. The length of the histogram represents the impact size of the different species (LDA score), and the different colors represent the species in different groups.

In LEfSe analysis, the nonparametric factorial sum-rank test was used to detect specific species relating significant abundance differences in different groups.  Figure 6(a) shows an evolutionary branch diagram of the 4 groups of sequences, with each circle representing the taxonomic level from the phylum to species.  Figure 6(b) shows 24 species with significant differences in abundance (LDA score > 4.0) between the 4 groups. At the genus level, the dominant members in group M were *Alloprevotella* and *Phascolarctobacterium*. *Alloprevotella* usually thought to be a harmful member in the intestinal flora [23], while *Phascolarctobacterium* thought to be related with the dysbiosis of intestinal microbiota structure in acute necrotizing pancreatitis rats [24]. The dominant member *Blautia* in group MS, as shown in Figure 5(b), may play an important role in disease relief, by responding to moxibustion and inflammation reduction [25]. Finally, one important biomarker at the genus level in group DS is *Enterococcus*, which is different from that of the MS group, suggesting that moxibustion and diclofenac sodium therapy have significantly different effects on intestinal flora.

## 4. Discussion

The two acupoints Dubi (ST35) and Zusanli (ST36) used in this study are located in the knee area and are widely used in the clinical treatment of KOA [26]. Acupuncture at ST35 has been demonstrated to be able to ameliorate KOA-related pain and reduce joint destruction [27]. The acupoint ST36, which is thought to be the representative point of the stomach meridian, is usually used for the treatment of disease with symptoms in the four limbs (distal joints) [28]. Our previous clinical studies have shown that the strategy of 30 min each treatment time for 4 weeks is effective and safe. Other studies also pointed out that the treatment time and length of each treatment is common use in many cases [29]. The results in this study suggest that moxibustion treatment in the MS group significantly alleviated cartilage histopathological damage, and the effect was equal to that of diclofenac sodium in the DS group. Similar results were obtained in a systematic evaluation [30], which showed that moxibustion was better than diclofenac sodium at improving the symptoms of patients with KOA. These results suggest that moxibustion provides a promising alternative therapy for KOA treatment. In addition, because of the close relationship between tissue damage and inflammation [3], we further analyzed whether moxibustion affects local and systemic inflammation during KOA treatment. Therefore, the above results showed that moxibustion may play a role in the treatment of KOA by alleviating local and systemic inflammation. Moxibustion treatment works on the local area and further alleviates systemic inflammation, which in turn is beneficial to the recovery of local cartilage injury.

There is more and more evidence in humans and rodents that microbiome is an important part of the whole life cycle and has an important impact on individual health [31]. In this study, it is worth noting that although there was no significant difference between the MS and DS groups in the treatment effect on the knee joint cartilage injury and inflammatory factors, there was a significant difference between the two groups on the impact on the intestinal flora. For the *α* index of the intestinal flora, it had good maintenance in the MS group while had tremendous changes in the DS group. The different intestinal flora in the DS group may due to the increase in the intestinal permeability, enteric bacterial numbers, and intestinal villous damage caused by nonsteroidal anti-inflammatory drugs such as diclofenac sodium [32, 33]. Therefore, the tremendous change suggested that diclofenac sodium has a destructive effect on host intestinal flora. Generally speaking, increased species richness in the intestinal flora produces an increased diversity of nutrient metabolism and increased resistance to harsh environments [34, 35]. The maintenance of the intestinal flora may also play an important role in host health. Thus, the moxibustion treatment strategy adopted in this study did not change the *α* diversity of intestinal flora significantly, and its mode of action may be the regulation of specific species of the intestinal microorganisms.

Previous studies have shown that many members of the intestinal flora can affect the physiological state of the host [36], and the homeostasis of the intestinal flora may be interrupted due to the occurrence of some diseases. Therefore, we annotated the OTUs obtained by sequencing and analyzed the composition of the intestinal flora at the phylum and genus levels. For the phylum level, in group M, excessive Gram-negative bacteria may increase the LPS content in the intestine or even the circulatory system, and many Proteobacteria are opportunistic pathogens in many cases [37]. As mentioned earlier, LPS plays an important role in KOA. For example, an increase in the LPS level can trigger an inflammatory innate immune response in the joint through TLR-4, leading to low-level inflammation [38]. In addition, studies have shown that the concentration of bacterial lipopolysaccharide in the serum and synovial fluid is positively correlated with the inflammatory response and severity of the joint inflammation [2]. Therefore, moxibustion may reduce the LPS level in the body by reducing the proportion of Gram-negative bacteria in the intestinal flora, thus alleviating the systemic inflammation of patients with KOA. A study of gut microbiota transplanting showed that the microbial community with high Firmicutes/Bacteroidetes ratio in the gut may increase energy extracted from the diet [39]. The decreased Firmicutes/Bacteroidetes ratio caused by high-fat diets was reported to cause mild inflammations [40]. Therefore, in this study, the increased Firmicutes/Bacteroidetes ratio may have a positive effect on inducing the anti-inflammatory response in joints. The effect also observed in a collagen-induced arthritis (CIA) mice model [41]. For the genus level, it has been reported that *Lactobacillus* and *Blautia* are probiotics in the intestine. *Lactobacillus* can inhibit or reduce the invasion of pathogenic bacteria and the production of intestinal inflammatory factors through adhesion and competition [42]. *Blautia* is an important Gram-positive bacterium that maintains intestinal homeostasis and can produce butyric acid and other beneficial metabolites and prevent inflammation by upregulating the proliferation of intestinal Treg cells [25]. Interestingly, the abundance of *Blautia* was also increased slightly in group M, which may be a response to the increased inflammatory state in group M, but the mechanism is not clear. The increase in *Shigella* abundance may aggravate the inflammation of the intestinal epithelium, even producing ulcers, followed by bloody or mucinous diarrhea. This is also consistent with the fact that NSAIDs damage the digestive tract. Therefore, the above results indicate that, moxibustion may alleviate the systemic inflammation of KOA by increasing the abundance of probiotic bacteria such as *Lactobacilli* and *Blautia*. Moreover, compared with the DS treatment group, the moxibustion treatment group did not have an enrichment of harmful bacteria, such as *Escherichia-Shigella*, making it a more beneficial treatment for host health.

By using the LEfSe method, to determine the members of the intestinal microflora directly regulated by moxibustion, we analysis the biomarkers with significant differences between the groups. For the biomarkers, in patients with rheumatoid arthritis, the accumulation of *Alloprevotella* is positively correlated with the biomarker of the inflammatory response C-reactive protein [23]. Other studies also showed that *Phascolarctobacterium* has an improvement effect on nonalcoholic fatty liver disease [43], and it is enriched in the synovial tissue of patients with KOA, but the specific role in arthritis has not been studied [44]. Therefore, the functions of these two biomarkers are complex and need further study. For the biomarker in the MS group, *Blautia* has been reported to an important role in maintaining intestinal homeostasis and producing butyric acid and other beneficial metabolites [25]. The analysis suggested that *Blautia* would play an important role in the treatment effect of moxibustion. Studies have shown that *Enterococcus* can synthesize short chain fatty acids such as butyric acid to have an anti-inflammatory effect in vivo [45, 46]. However, others [47] have summarized the relevant reports of *Enterococcus* and concluded that the strain is a potential probiotic and an emerging pathogen that can produce virulence factors and drug resistance. Therefore, in the DS group, the effect of *Enterococcus* as a biomarker of the intestinal flora needs further study.

## 5. Conclusions

Moxibustion has been used for a long time in the treatment of knee arthritis and has a significant effect, but the mechanism is not fully understood. In this study, we used high-throughput sequencing of the 16S rRNA gene of the intestinal flora to explore the regulatory effect of moxibustion on the intestinal flora during the treatment of KOA and to investigate the possible relationship between changes in the flora and inflammation. The results showed that moxibustion regulated the community structure of the intestinal flora and obviously increased the abundance of probiotics such as *Blautia*. The analysis showed that this regulatory effect has a positive impact on knee osteoarthritis. Therefore, compared with diclofenac sodium, moxibustion is a gentler treatment for individual health. In conclusion, we suggest that moxibustion can affect knee joint cartilage and reduce the degree of KOA cartilage damage by regulating the composition of the intestinal flora to alleviate inflammation in rats. However, there are still some deficiencies in this study. For example, we did not study the “dose-response” relationship using multiple moxibustion doses, which is not conducive to an in-depth discussion of the relationship between moxibustion and the change in the intestinal flora. The mechanism by which moxibustion reduces the expression of proinflammatory cytokines during the regulation of inflammation is still unknown. Moreover, the lack of inflammatory and intestinal flora baseline measurement also is the limitation of this study. However, increasing evidence of the relationship between the disease process of KOA and the imbalance of the intestinal flora makes the intestinal flora a very promising new target for KOA treatment.

## Figures and Tables

**Table 1:** Modified Mankin score.

Structure		Cell count		Staining	
Intact	0	Normal	0	Normal	0
Irregular surface	1	Replicating	1	Slightly reduced	1
Fissures to the transitional zone	2	Hypercellularity	2	Moderately reduced	2
Fissures to the deep zone	3	Hypocellularity	3	Severely reduced	3
Complete disorganization	4			Absence of dye	4

**Table 2:** The *α* diversity of the intestinal flora.

Group	ACE	Chaol	Simpson	Shannon
N	418.07 ± 16.91	423.79 ± 23.72	0.07 ± 0.04	3.80 ± 0.60
M	450.94 ± 19.11	458.50 ± 22.47	0.03 ± 0.01^*∗*^	4.40 ± 0.05
MS	447.58 ± 32.02	450.73 ± 30.93	0.05 ± 0.02	4.12 ± 0.31
DS	397.78 ± 7.14^#^	401.38 ± 16.19^#^	0.09 ± 0.05^#^	3.30 ± 0.73^#^

Compared with N, ^*∗*^*P* < 0.05. Compared with M, ^#^*P* < 0.05.

## Data Availability

The data used to support the findings of this study are available from the corresponding author upon request.
